# In Situ Green Synthesis of Co_3_O_4_@ZnO Core-Shell Nanoparticles Using *Datura stramonium* Leaf Extract: Antibacterial and Antioxidant Studies

**DOI:** 10.1155/2023/5019838

**Published:** 2023-11-29

**Authors:** Gezahegn Tadesse, H. C. Ananda Murthy, C. R. Ravikumar, T. Naveen Kumar, Lema Teshome, Tegene Desalegn

**Affiliations:** ^1^Department of Applied Chemistry, School of Applied Natural Science, Adama Science and Technology University, Adama, P.O. Box 1888, Ethiopia; ^2^Department of Prosthodontics, Saveetha Dental College & Hospital, Saveetha Institute of Medical and Technical Science (SIMATS), Saveetha University, Chennai 600077, Tamil Nadu, India; ^3^Research Centre, Department of Science, East West Institute of Technology, Bangalore 560091, Karnataka, India; ^4^Department of PG-Chemistry, Surana College Autonomous, Affiliated Bangalore University, Bangalore 560004, India; ^5^Research Institute of Materials Chemistry, Chungnam National University, Daejeon 34134, Republic of Korea

## Abstract

Investigating and synthesizing potent antibacterial NPs using biological methods is highly preferred, and it involves nontoxic, cost-effective, and environmentally friendly chemicals and methods. Antibiotic drug resistance and oxidative stress have become a serious public health issue worldwide. Hence, the key objective of this study was to biologically synthesize and characterize the potent antibacterial Co_3_O_4_@ZnO core-shell nanoparticles for the antibacterial application. The radical scavenging ability of green synthesized Co_3_O_4_@ZnO core-shell nanoparticles was also determined. In this study, Co_3_O_4_@ZnO core-shell nanoparticles (CZCS NPs) have been synthesized using three different core to shell materials ratios of Co_3_O_4_ to ZnO (0.5 : 0.25 CZCS (1), 0.5 : 0.5 CZCS (2), and 0.5 : 0.75 M CZCS (3)) by employing *Datura stramonium* leaf extract. The polycrystalline nature of Co_3_O_4_@ZnO core-shell nanoparticles was investigated using the XRD and SAED characterization techniques. The investigated nanostructure of Co_3_O_4_@ZnO core-shell nanoparticles appeared with Co_3_O_4_ as the core and ZnO as an outer shell. Additionally, a variety of physicochemical properties of the nanoparticles were determined using various characterization techniques. The average crystallite sizes of CZCS (1), CZCS (2), and CZCS (3) were found to be 24 ± 1.4, 22 ± 1.5, and 25 ± 1.5 nm, respectively. The band gap energy values for CZCS (1), CZCS (2), and CZCS (3) determined from the UV-DRS data were found to be 2.75, 2.76, and 2.73 eV, respectively. The high inhibition activities against *S. aureus*, *S. pyogenes*, *E. coli*, and *P. aeruginosa* bacterial strains were obtained for the small size CZCS (2) nanoparticles at the concentration of 100 mg/mL with 22 ± 0.34, 19 ± 0.32, 18 ± 0.45, and 17 ± 0.32 mm values, respectively. The high inhibition performance of CZCS (2) nanoparticles against Gram-positive and Gram-negative bacteria which is even above the control drug ampicillin is because of its small size and synergistic effect. The percentage scavenging activity of Co_3_O_4_@ZnO core-shell nanoparticles was also studied and CZCS (2) nanoparticles showed a good scavenging capacity (86.87%) at 500 *μ*g/mL with IC50 of 209.26 *μ*g/mL.

## 1. Introduction

The current development of nanotechnology has encouraged researchers in the field to design and investigate novel nanomaterials with properties that significantly address the increasing demand for their application towards biological and environmental remediation. Among the recently fabricated nanomaterials, metal nanoparticles (NPs) are highly interesting, due to their unique and modified physicochemical properties, such as catalytic activity, stability, size, shape, surface energy, reactivity, surface area, sensitivity, chemical composition, porosity, crystallinity, electrical, magnetic, and optical [1–4]. The particle size and surface area to volume ratio of NPs are the vital properties that determine other characteristics of the NPs. Researchers have reported the use of various methods and approaches to develop metal NPs with a small size and a large surface area to volume ratio [5–8]. For more than half a century, single NPs have been studied widely. However, over the past few decades, hybrid NPs such as core-shell structures have become more attractive from a technological viewpoint associated with the unique properties that enable them to be used in several applications [9–11]. In core-shell NPs, one or more core materials are coated by the other organic or inorganic shell materials which enhance the properties of the core materials [12–14].

The core-shell nanostructures are preferable due to the synergistic effect of the core and shell materials [15]. The core-shell metal oxide nanostructures are highly functional with enhanced physicochemical properties. The core-shell nanostructures have unique properties such as thermal stability, biocompatibility, selectivity, dispersibility, and reactivity than those of their single nanoparticle counterparts [16, 17]. The biggest advantage of core-shell NPs is enhancing the low potential that is not well possessed by single NPs and minimizing the side effects of their parent materials. As reported in some of the studies, even at high concentrations, core-shell NPs were almost nontoxic, as compared to the corresponding monometallic NPs [18–22].

Among inorganic oxides, Co_3_O_4_ is an attractive oxide because of its mechanical strength, availability, and its cost in contrast to other noble materials such as Ag, Au, and Pt [23–25]. The nanostructure of Co_3_O_4_ has several properties that enable it to be used in different fields of study. The special characteristics of Co_3_O_4_ NPs such as catalytic properties, sensors, magnetic behavior, bioavailability, storage, and reactivity attract the intention of numerous researchers. The high surface area because of its small size empowers it to be used in a variety of applications such as photocatalysts, capacitors, field emission materials, sensing, drug delivery, magnetic resonance imaging, biomedical, and antimicrobial agents [26–28]. Co_3_O_4_, a white powder having a band gap of 1.48 eV, is an antiferromagnetic p‐type semiconductor. It has a spinel crystal structure and is widely used in different applications and is developed as the core nanostructure [29, 30]. In addition to cobalt oxide NPs, zinc oxide nanostructure has also received the attention of researchers. Due to their unique properties, ZnO NPs are at the crown of the research and widely used as a pigment, ointment, adhesive, food, cosmetics, sunscreen, lubricants, paints, etc. The special characteristics of ZnO NPs are bioavailability and low toxicity to normal cell, which promote its activity in disease treatment such as cancer, oxidative stress, bacterial infections, and diabetes [28, 31, 32].

Additionally, ZnO NPs have good antibacterial property and are widely used in drug delivery. The n-type semiconductor ZnO has a large band gap than Co_3_O_4_ NPs with a value of 3.37 eV and is known for its unique properties such as bioavailability, biocompatibility, low toxicity, and high solubility. Due to their novel properties, the hybridized structure called core-shell nanoparticles of Co_3_O_4_ and ZnO NPs has been designed to investigate their synergistic effects specifically on antibacterial strains. Based on their novelty and physicochemical properties, Co_3_O_4_ has been designated as the core of the nanoparticles whereas ZnO NPs serves as the shell in this particular study [24, 33, 34].

In Co_3_O_4_@ZnO core-shell NPs, the core material Co_3_O_4_ was coated by ZnO which acts as a shell to control its reactivity and enhance thermal stability. Additionally, the shell decreases the toxicity of Co_3_O_4_ NPs and increases the dispersibility [35–37].

**Figure 1:**
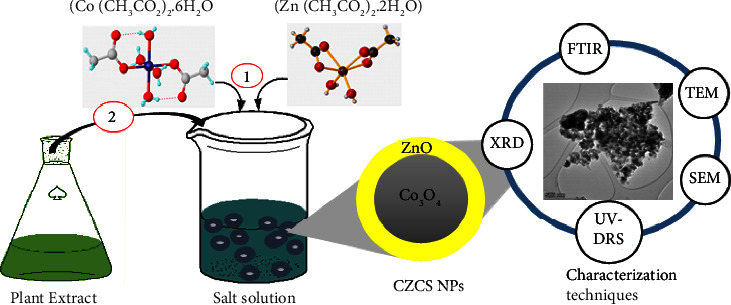
A schematic presentation of *in situ* green synthesis of Co_3_O_4_@ZnO core-shell NPs.

As mentioned in previous reports, several physical and chemical techniques were used to prepare metal oxide nanoparticles. The top-down is when particles are broken down from macro to nanoscale and bottom-up builds up the nanoparticles from atoms and molecules. The bottom-up method is more chosen because of the difficulties in achieving a uniform shape, desired size, and perfect surface of nanoparticles [38–40]. Nowadays, the most widely used techniques in the synthesis of the nanoparticle are chemical methods and biological methods. The most common chemical methods such as vapor deposition, sol-gel, thermal evaporation, microwave-assisted, pulsed laser deposition, electrochemical reaction, sputtering, hydrothermal, solvothermal, and microemulsion are used in the fabrication of nanoparticles [41–44]. Similarly, Co_3_O_4_@ZnO core-shell NPs have been synthesized by using the chemical and physical methods. The chemically synthesized Co_3_O_4_@ZnO core-shell NPs appeared to possess a particle size of 12 nm and had a band gap value of 4.97 eV which was larger than their parent materials. The hydrothermal/sol-gel method synthesized Co_3_O_4_@ZnO core-shell NPs with an average inner core material size of 22 nm, and outer shell with 56 nm particle size from the TEM image was also reported [17, 45–47]. However, chemically synthesized Co_3_O_4_@ZnO NPs are not environmentally friendly but affect both human and environmental health. Green synthesis methods for the preparation NPs has become a promising alternative to chemical methods because they require low energy, involve low cost, low temperature, and low pressure, are environmentally friendly, and have a one-pot synthesis. Green synthesis route involves the use of different parts of plants, bacteria, fungi, and algae. The biomolecules present in these sources are used to reduce and stabilize the NPs by preventing the agglomeration of grains during the preparation of NPs [48–50]. In this work, the green synthesis method which is an environmentally friendly, cost-effective, and nontoxic method in which *Datura stramonium* leaf extract has been applied for the synthesis of Co_3_O_4_@ZnO core-shell NPs as illustrated in Figure 1.

*Datura stramonium* is a flowering weed commonly called Astenager in Amharic or Menji in Afaan Oromo and it belongs to the family *Solanaceae*. Several phytochemical constituents of *Datura stramonium* such as alkaloids, flavonoids, tannins, saponins, phenol, proteins, glycosides, and steroids have been studied. Phytochemicals such as alkaloids, flavonoids, tannins, saponins, terpenoids, and steroids found in *Datura stramonium* leaf were extracted using petroleum ether, chloroform, and methanol solvents, and the positive screening test results obtained confirmed the presence of the aforementioned phytochemicals [51–53]. There exists a report of the development of a series of facilely accessible quinoline derivatives that display potent antibacterial activity against a panel of multidrug-resistant Gram-positive bacterial strains, especially *C. difficile* [32, 54, 55]. However, there is no report regarding the use of *Datura stramonium* plant extract for the synthesis of NPs.

Nowadays, bacterial infection and oxidative stress have become priorities among the global health threats. The bacterial infectious diseases are caused by the potential microorganisms that are unfriendly to human beings [56, 57]. Recent reports have revealed that the bacterial resistance against the antibiotics has become a serious health issue, posing a global risk [58]. The lack of development of new drugs for the bacteria-caused diseases, features of multidrug-resistant bacteria, and misusing antibiotics enable the bacteria to be resistant to the drug [59–61]. It is estimated that by 2050, antibiotic resistance will have caused approximately 300 million deaths, with an economic loss of $100 trillion, and according to the World Health Organization (WHO) report, antibiotic resistance is one of the major health problems of the century. These concerns have initiated a search for innovative strategies in antimicrobial therapies [62]. Among the strategies that have been under investigation, the use of antimicrobial peptides, phage therapy, therapeutic antibodies, quorum sensing inhibitors, and antimicrobial NPs could be mentioned [54, 63]. As alternatives for overcoming the problems due to drug-resistant bacteria, several NPs such as CuO, ZnO, NiO, Ag, TiO_2_, and Co_3_O_4_ NPs have been widely studied [64, 65]. The advantage of using NPs over conventional drugs is the high efficacy in treatment because of their small size, specificity, and low side effect on the host. In this work, the antibacterial activities of Co_3_O_4_@ZnO core-shell NPs have been studied against two Gram-positive (*S. aureus* ATCC25923 and *S. pyogenes* ATCC19615) and Gram-negative (*E. coli* ATCC25922 and *P. aeruginosa* ATCC27853) strains [66].

In addition to drug-resistant bacteria, the oxidative stress that is resulting from the disproportion between fabrication and accumulation of reactive oxygen species (ROS) has been noticed also as a critical health issue. In normal cellular respiration under both physiological and pathological conditions, mitochondria produce ROS such as superoxide (O_2_^•−^), singlet oxygen (^1^O_2_), hydrogen peroxide (H_2_O_2_), and hydroxyl radicals (•OH). Natural antioxidants such as superoxide dismutase (SOD), catalase (CAT), and glutathione peroxidase (GPx) found in the human body are known to scavenge ROS molecules to prevent the adverse effects on health [61, 66, 67]. However, the overproduction of ROS molecules induces oxidative stress. Hence, oxidative stress leads to several diseases such as cancer, diabetes, inflammation of different organs, and acute diseases. To scavenge ROS, a variety of conventional antioxidants and NPs such as Fe_2_O_3_, Ag, Cu, Co_3_O_4_, and ZnO NPs have been widely studied [68–71]. In this work, the antioxidant activities of biologically synthesized Co_3_O_4_@ZnO core-shell NPs were also studied.

## 2. Experimental Methods

### 2.1. Chemical and Reagents

The chemicals in this study were ethanol (99.9%, LabTech Chemicals), cobalt acetate hexahydrate (Co(CH_3_CO_2_)_2_.6H_2_O), distilled water, zinc acetate hexahydrate (Zn(CH_3_CO_2_)_2_.2H_2_O), Müller–Hinton agar, ampicillin, sodium hydroxide (NaOH) (Sigma-Aldrich), 2, 2-diphenyl-1-picrylhydrazyl (DPPH), ascorbic acid, dimethyl sulfoxide (DMSO), and methanol (CH_3_OH). The chemicals and reagents were all analytical grades and utilized without further purification.

### 2.2. Collection and Preparation of Plant Extract

**Figure 2:**
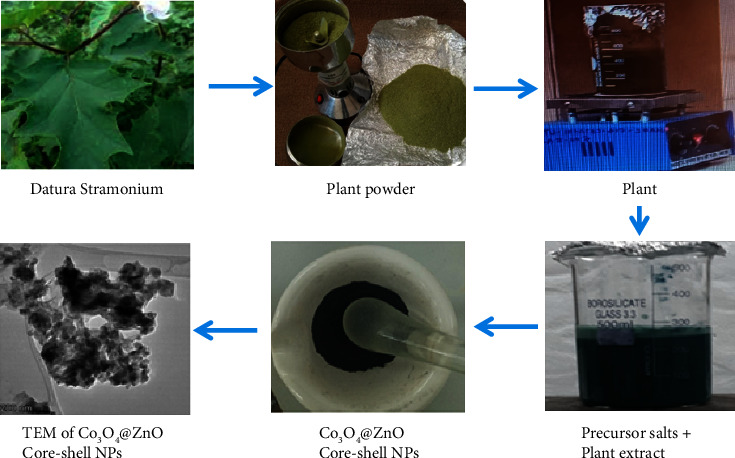
A schematic representation of the synthesis of Co_3_O_4_@ZnO core-shell NPs.

The leaves of the *Datura stramonium* plant used in this study were collected from around Adama Science and Technology University, East Shewa, Ethiopia. The specimen was identified and authenticated at the Addis Ababa University Herbarium (Voucher No. AUGH016) and documented for reference purposes along with the medicinal plants. The leaves were washed three times using distilled water to remove the dust particles from the surface of the leaves. The contaminant-free *Datura stramonium* leaves were dried under shadow at room temperature for 15 days and ground using a micro-grinding machine. 30 g of *Datura stramonium* leaf powder was added to a 1000 mL conical flask containing 500 mL distilled water. Using a hotplate, the mixture was heated to 40°C and stirred for 90 min. The filtrate was collected using Whatman Number 1 filter paper from the boiled colloidal solution cooled at room temperature. Finally, the filtrate was stored in the refrigerator at 4°C for the synthesis of the NPs. The procedure involved is presented in Figure 2.

### 2.3. Green Synthesis of Co_3_O_4_@ZnO Core-Shell NPs

**Figure 3:**
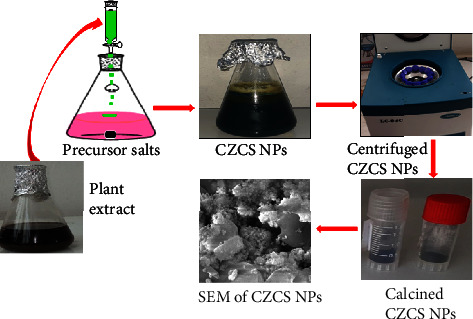
A schematic of the green synthesis of Co_3_O_4_@ZnO core-shell nanoparticles.

The Co_3_O_4_@ZnO core-shell NPs were synthesized using the *Datura stramonium* leaf extract from cobalt acetate hexahydrate (Co(CH_3_CO_2_)_2_.6H_2_O) and zinc acetate dihydrate (Zn(CH_3_CO_2_)_2_.2H_2_O) precursor salts by keeping the concentration of core/inner part material constant and varying the concentration of shell/outer material as shown in Figure 3. The volume of the two precursor salts and plant extract was constant throughout the synthesis. Three different concentration ratios of core to shell materials, namely, 0.5 : 0.25 CZCS (1), 0.5 : 0.5 CZCS (2), and 0.5 : 0.75 M CZCS (3), of Co_3_O_4_ to ZnO were considered during the synthesis of the NPs.

For instance, in the synthesis of CZCS (2), 100 mL of Plant leaf extract was gradually added to a 1000 mL beaker containing the mixture of 50 mL of 0.5 M (Co(CH_3_CO_2_)_2_.6H_2_O) and 50 mL of 0.5 M Zn(CH_3_CO_2_)_2_ and stirred about 4 h. Then, 0.1 M of NaOH was added (pH = 12) under stirring for 30 min resulting in the precipitation of the NPs. The precipitate was kept in a refrigerator overnight. The precipitate was centrifuged at 2000 rpm for 20 min and washed three times using ethanol and distilled water. The washed CZCS (2) was collected on a ceramic crucible and dried in an oven at 100°C and finally calcined at 360°C using a muffle furnace, and the obtained crystalline nanostructure was stored for the purpose of the characterization [44]. The same procedure was repeated for the synthesis of CZCS (1) and CZCS (3).

### 2.4. Antibacterial Activities

The antibacterial activities of the synthesized NPs, CZCS (1), CZCS (2), and CZCS (3), were evaluated using the disc diffusion method. The inhibition efficiency of the biologically synthesized Co_3_O_4_@ZnO core-shell NPs was studied for Gram-positive (*S. aureus* ATCC25923 and *S. pyogenes* ATCC19615) and Gram-negative (*E. coli* ATCC25922 and *P. aeruginosa* ATCC27853) bacterial strains. In the disc diffusion method, 12 g of broth agar was prepared in 200 mL of distilled water. The solution of nutrient agar was dispensed onto the Petri dish. The poured liquid nutrient agar was solidified on the Petri dish and well homogenized, and the grown culture of the four bacteria was inoculated and kept on a shaker at 35°C for 24 h at 200 rpm. The standard drug ampicillin (positive control) was used in the analysis for determining the antibacterial activities of biologically synthesized Co_3_O_4_@ZnO core-shell NPs. In addition to this, DMSO was used as solvent and negative control. Then, the biologically synthesized CZCS (1), CZCS (2), and CZCS (3) were applied to the Gram-positive and Gram-negative strains in four different concentrations (25, 50, 75, and 100 mg/mL). The plates were incubated at 37°C for about 24 h and checked for the zone of inhibition. The scale of the image was determined in millimeters using ImageJ software.

### 2.5. Antioxidant Activities

The stable purple 2, 2-diphenyl-1-picrylhydrazyl (DPPH) free radical was utilized to investigate the radical scavenging ability of biologically synthesized CZCS NPs. The activity was measured following the modified procedure used in the previous work report [54, 72]. A 4 mL of 100 *μ*M DPPH was prepared in DMSO and added to methanolic 1000 *μ*L CZCS NPs with 50, 100, 200, 300, and 500 *μ*g/mL. The mixture was sonicated and kept in the dark chamber for 30 min, followed by incubation at 37 ± 2°C for the same time. The UV-vis absorbance of positive control (ascorbic acid) of the same concentration, DPPH, and the incubated sample was measured at 517 nm. All the experiments were performed in triplicate, and the average absorbance for each sample was considered. Finally, the percentage scavenging capacity of NPs was determined using the following equation [73].

(1) $$\begin{array}{c} \%\text{ Radical scavenging activity}=\frac{\text{Absorbance of control}-\text{Absorbance of sample}}{\text{Absorbance of control}}*100. \end{array}$$

### 2.6. Characterization

The successful synthesis and biological applications of *Datura stramonium* leaf extract-mediated Co_3_O_4_@ZnO core-shell NPs have been confirmed by using different characterization techniques such as thermogravimetric analysis/differential thermal analysis (TGA/DTA), Ultra Violet-differential reflectance spectroscopy (UV-DRS), X-ray diffraction (XRD), Fourier-transform infrared (FT-IR), scanning electron microscopy-energy dispersive spectroscopy (SEM-EDAX), and transmission electron microscopy-high resolution transmission microscopy (TEM-HRTEM). The thermal stability of CZCS (2) was determined using thermogravimetric analysis/differential thermal analysis (TGA-DTA, DTG-60H). The band gap, the crystallinity, and the functional group of the synthesized NPs were determined using the Ultra Violet-diffused reflectance spectroscopy (UV-DRS), X-ray diffraction (XRD), and Fourier-transform infrared (FT-IR), respectively. The scanning electron microscopy-energy dispersive spectroscopy (SEM-EDS) was used to determine the morphology and elemental composition of the NPs. The transmission electron microscopy-high resolution transmission microscopy (TEM-HRTEM) was used to illustrate the inner details of microstructure of CZCS NPs [74].

## 3. Result and Discussion

### 3.1. Phytochemical Analysis

In this study, ten selected phytochemical constituents of *Datura stramonium* leaf were screened using the methods mentioned in Table 1. The phytochemical screening showed a positive result. Among the screened phytochemicals, only steroids show negative tests [52]. The selected phytochemicals are mostly phenolic and carboxylic groups containing molecules which are used to reduce the metal to its nanoscale size. In addition to this, these bioactive molecules are utilized as a capping agent to prevent the agglomeration of nanoparticles during synthesis [75].

### 3.2. Thermal (TGA/DTA) Analysis

**Figure 4:**
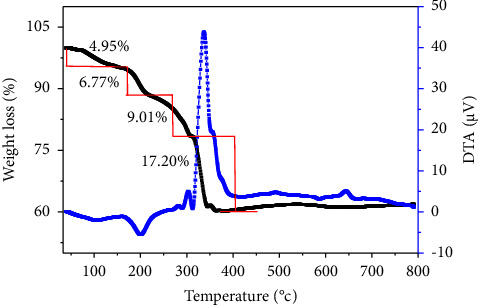
The TGA/DTA plots of Co_3_O_4_@ZnO core-shell NPs.

The thermal stability of CZCS (2) sample is presented as shown in Figure 4. The TGA-DTA plots showed the degradation pattern of the NPs and were used to determine the calcination temperature at which synthesized NPs become thermally stable. The percentage decomposition of several chemical constituents has been determined from the curve of TGA. The endothermic and exothermic energy changes of the sample were determined by DTA.

The change in physical properties of the CZCS (2) was monitored as a function of controlled temperature change. In this study, 10 mg of CZCS (2) was analyzed within a temperature range of 29–800°C and the sample started to decompose as the temperature rose. At a temperature of 360°C and onwards, the core-shell nanostructures became stable. Some molecules such as ethanol, water, and organic bioactive molecules from the plant extract were decomposed before the calcination point of core-shell nanostructures. The TGA curves of CZCS (2) which run at a heating rate of 10°C min^−1^ have shown the decomposition change at four different steps. The first two weight losses, 4.95 % and 6.77 %, recorded were due to the decomposition of ethanol and water, respectively, whereas, the remaining two steps (9.01 and 17.2%) were due to the loss of organic molecules from the plant extract. In general, about 37.93% weight of the sample was lost in the thermal decomposition of CZCS (2) within the temperature range of 30–360°C. The difference in steps of weight loss is because of the gelly-like property of CZCS (2) NPs which highly incorporate organic molecules and decomposed at different temperatures. This corroborated that CZCS (2) was reduced and capped by the phytochemicals of the plant extract [56]. As shown in Figure 4, from the TGA/DTA curve, it can be noted that above a temperature of 360°C, CZCS (2) NPs were found to be thermally stable.

### 3.3. X-Ray Diffraction (XRD) Analysis

**Figure 5:**
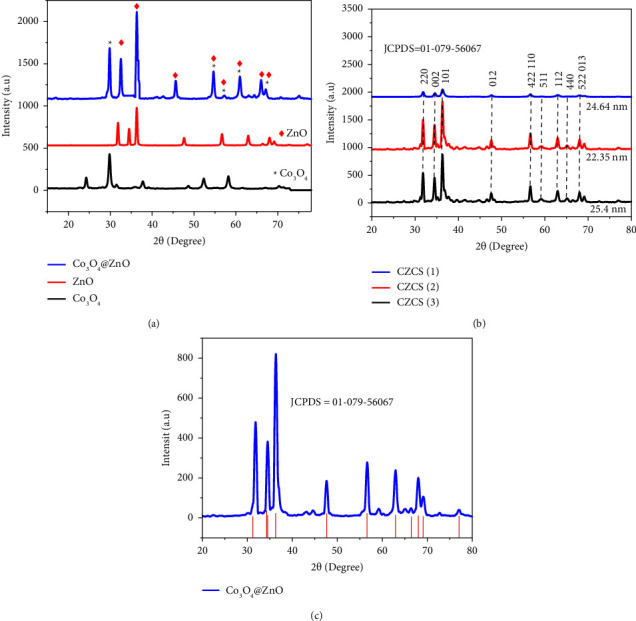
The X-ray diffraction patterns of (a) Co_3_O_4_@ZnO core-shell NPs with parent ZnO and Co_3_O_4_ NPs, (b) CZCS (1), CZCS (2), and CZCS (3) NPs, and (c) riveted refinement of Co_3_O_4_@ZnO NPs.

The X-ray diffraction (XRD) patterns of Co_3_O_4_@ZnO core-shell NPs are shown in Figure 5. The XRD patterns of Co_3_O_4_@ZnO, Co_3_O_4_, and ZnO NPs are shown in Figure 5(a), and Figure 5(b) depicts the diffraction patterns of NPs of various ratios of precursors, CZCS (1), CZCS (2), and CZCS (3). In all the NPs, the concentration of Co_3_O_4_ core nanoparticle was kept constant throughout the work. In XRD analysis, the electron of both the core and shell nanostructure scattered the X-rays and produced different constructive interference patterns with different intensities. The diffraction peaks observed in separate Co_3_O_4_ NPs and ZnO NPs have appeared in the biosynthesized core-shell NPs which are consistent with other previous reports. The less intense diffraction peaks were observed at 31.86, 56.66, 59.12, 65.14, and 68.06 of 2*θ* values which were assigned to the crystal planes with miller indices (220), (442), (511), (440), and (522), respectively, which correspond to Co_3_O_4_ NPs [73].

The intense peak of ZnO shell NPs was seen at 2*θ* values of 34.52, 36.34, 47.71, 56.68, 62.96, and 68.06 which correspond to (002), (101), (012), (110), (112), and (013) planes, respectively. As shown in Figures 5(a) and 5(b), all the diffraction peaks of the Co_3_O_4_@ZnO core-shell NPs within CZCS (1), CZCS (2), and CZCS (3) have fitted the peaks of the corresponding parent particles which is similar to the previous work report [29].

In addition to this, the formation of all CZCS (1), CZCS (2), and CZCS (3) correctly matched the standard database of JCPDS card no. 01-079-5606. The average crystalline size of *in situ* green synthesized Co_3_O_4_@ZnO core-shell NPs has been calculated using the following formula:

(2) $$\begin{array}{c} D=\frac{k\lambda }{\beta \;Cos\theta }, \end{array}$$

where *D* stands for average crystallite size (nm), *k* is Scherrer's constant with a 0.154 nm value, *λ* is the wavelength of the X-ray source which is CuK *α* with 1.5406 Å value and *β* is the full-width at half-maximum (FWHM) of the diffraction peak appeared at 2*θ* in radian, and *θ* is half of the angle between transmission and diffraction [52]. The average crystal sizes of CZCS (1), CZCS (2), and CZCS (3) were found to be 24 ± 1.4, 22 ± 1.5, and 25 ± 1.5 nm, respectively. Relatively, the calculated average crystalline size of CZCS (2) was found to be 22 ± 1.5 nm which is smaller than the two ratios. The difference in the average crystalline size of Co_3_O_4_@ZnO core-shell NPs is due to the variation in ZnO NPs in their concentration. In the case of CZCS (1), the excess amount of phytochemicals present in the leaf extract is believed to compete with each other than reduce the NPs. But, in CZCS (3), the number of phytochemicals present in the extract was believed to be less enough to reduce and cap the NPs which resulted in the agglomeration of NPs.

Additionally, the % error in average crystallite size of CZCS (1), CZCS (2), and CZCS was calculated using the following equation:

(3) $$\begin{array}{c} \%\text{ error}=\left(\frac{\left|\text{estimated value}-\text{real}\text{ value}\right|}{\text{real value}}\right)*100. \end{array}$$

The real average crystallite size value of CZCS (1), CZCS (2), and CZCS (3) was found to be 24.64, 22.35, and 25.4 nm for CZCS (1), CZCS (2), and CZCS (3), respectively. The calculated % error average crystallite size of the green synthesized CZCS (1), CZCS (2), and CZCS was 1.4, 1.5, and 1.5%, respectively. This indicates that CZCS (1), CZCS (2), and CZCS had average crystallite sizes of 25 1.4, 22 1.5, and 25 1.5 nm, respectively.

### 3.4. FT-IR Spectral Analysis

**Figure 6:**
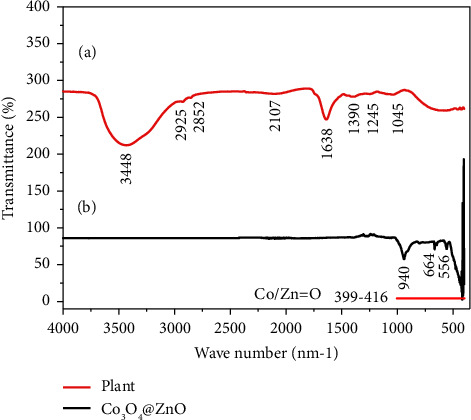
FTIR spectra of (a) plant leaf powder and (b) Co_3_O_4_@ZnO core-shell NPs.

The variety of *Datura stramonium* functional groups of plant leaf powder and that of the synthesized NPs were determined using FTIR. The FTIR analysis indicated the functional groups of different phytochemical constituents that were involved in the reduction during the synthesis of core-shell nanostructures. As shown in Figure 6(b), the functional groups seen in the FTIR spectra of the plant have not appeared in the spectra of Co_3_O_4_@ZnO core-shell NPs. The absence of functional groups in the spectra of Co_3_O_4_@ZnO core-shell NPs indicated that these functional groups were used in the reduction process. As depicted in Figure 6(a), in the spectra of plant powder, a variety of functional groups absorbed the IR radiation at 3448, 2925, 2852, 2107, 1637, 1390, 1245, 1043, and 556 cm^−1^. Similarly, the *Datura stramonium* leaf extract-mediated core-shell nanostructures have shown absorption bands at 940, 664, 556, and 399.416 cm^−1^.

The broad absorption band at ∼3448 cm^−1^ is believed to be due to the stretching of O-H of a phenolic group. The absence of this broad absorption band in the spectra of NPs was due to the calcination of CZCS (2) at a high temperature that degrades and eliminates. The spectral bands observed at 2925 and 2852 cm^−1^ were because of C−H stretching vibration of alkane and alkene groups, respectively [76]. The bands that appeared at 2107, 1637, and 1390 cm^−1^ were due to the presence of C ≡ C, C = C, andC = N functional groups, respectively. Additionally, the absorption bands of C-O vibrations of the ester group and the C-N vibration of amide groups were found at 1245 and 1045 cm^−1^, respectively [51, 77].

In FTIR analysis of Co_3_O_4_@ZnO core-shell NPs, almost all the functional groups present in the phytochemicals of *D. stramonium* leaf powder were not seen. Figure 6(b) depicts the absorption band formed as a result of the stretching vibration of Zn-OH. The stretching modes of Zn-O were also found at 940 and 399.98 cm^−1^. The analysis also showed O-Co-O and Co-O stretching vibration at 664 and 556 cm^−1^, respectively.

### 3.5. UV-DRS Analysis

**Figure 7:**
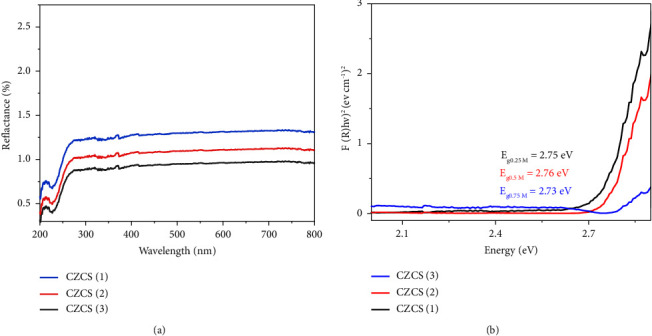
The reflectance percentage (a) and (b) band gap energy values of Co_3_O_4_@ZnO core-shell NPs.

UV-diffused reflectance spectroscopy has been used to determine the optical band gap energy of Co_3_O_4_@ZnO core-shell NPs. As depicted in Figures 7(a) and 7(b), the percentage of reflectance and optical band gap energies (*E*_g_) of biologically synthesized Co_3_O_4_@ZnO core-shell NPs have been found, respectively. The band gap energy of CZCS (1), CZCS (2), and CZCS (3) was calculated by converting the generated reflectance using the Kubelka–Munk formula [74].

(4) $$\begin{array}{c} F\left(R\right)=\frac{{\left(1-R\right)}^{2}}{2R}, \end{array}$$

where *F* (*R*) is equivalent to absorption values and *R* is diffuse reflectance.

As shown in Figure 7(b), the direct band gap energy from the Tauc plot for CZCS (1), CZCS (2), and CZCS (3) was deduced to be 2.75, 2.76, and 2.73 eV, respectively. These values were obtained from the plot of (*F*(*R*)*hν*)^2^ versus 1240/*λ*eV by extrapolating within the linear range of the graph. The *E*_g_ value of CZCS (2) is greater than CZCS (1) and CZCS (3) due to its small size which matches with XRD results and assured that the smaller the nanosize is, the larger the band gap energy will be. Additionally, the *E*_g_ values of all the three ratios showed smaller values than their bulk parents [78].

### 3.6. SEM-EDAX Analysis

**Figure 8:**
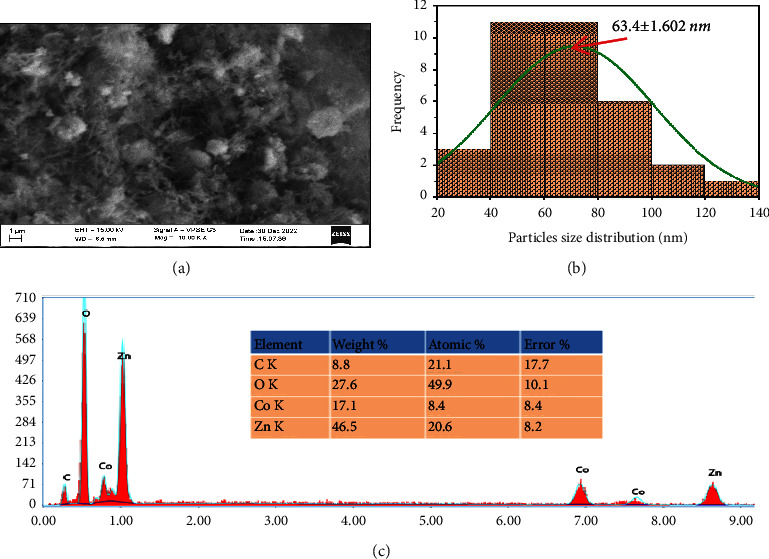
SEM image of (a) *Datura stramonium* leaf extract-mediated Co_3_O_4_@ZnO NPs, (b) particle size distribution, and (c) elemental composition Co_3_O_4_@ZnO NPs obtained from EDAX.

The shape and size of Co_3_O_4_@ZnO core-shell NPs were analyzed using scanning electron microscopy (SEM). In scanning electron microscopy, a packet of the electron beam interacts with the surface of samples and the shape of CZCS NPs was determined from the scanned surface.  Figure 8(a) depicts the rod-like and spherical shape of Co_3_O_4_@ZnO core-shell NPs. The spherically shaped NPs are highly branched from the same point which seems most likely shell material that surrounded the core NPs. The overall shape of CZCS NPs shows agglomeration due to the adhesion force developed from the electrostatic interaction between the oxygen of core NPs and hydroxide of shell NPs. From SEM images, the diameters of the particles were generated in nanometers using ImageJ software. As shown in Figure 8(b), the average crystalline size of the particles was found to be 63.40 ± 1.602 nm.

The elemental composition of Co_3_O_4_@ZnO NPs was also identified using energy dispersion X-ray spectroscopy within the 0 to 10 keV range. From the EDAX analysis indicated in Figure 8(c), the synthesized NPs were found to contain Co, Zn, O, and C elements. An intense peak of ∼ 500 at 1 KeV was attributed to the elemental composition of shell NPs whereas those making up the core NPs exhibited ∼100 counts at 0.8 KeV. As an impurity, carbon element is believed to be from three different sources. Most likely, it is from the atmosphere in the form of carbon dioxide which is absorbed on the surface of NPs, formed during the calcination of synthesized materials in a muffle furnace, and from specimen holders which were used for coating nonconducting materials for analysis. The atomic percentages of Co, Zn, and O were found to be 8.4, 20.6, and 49.9%, respectively. The atomic percentage of shell NPs is more than twice the core NPs for the materials synthesized from the equal molarity of precursor salts. The weight percentage Co, Zn, and O obtained from EDAX was 17.6, 46.5, and 27.6%, respectively.

### 3.7. TEM/HR-TEM and SAED Analysis

**Figure 9:**
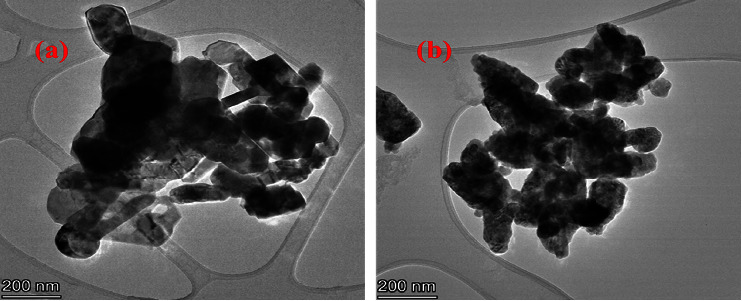
The TEM image of Co_3_O_4_ NPs (a) and ZnO NPs (b).

The cuboid and spherical shapes were obtained for Co_3_O_4_ NPs and the ZnO NPs from the TEM analysis, as shown in Figures 9(a) and 9(b), respectively. The average particle sizes of Co_3_O_4_ and ZnO NPs were found to be 15 nm and 17 nm, respectively. The Co_3_O_4_@ZnO NPs were characterized by using TEM/HR-TEM technique. In TEM/HR-TEM, the detailed analysis was carried out to investigate the shape, structure, size, and particle size distribution of the NPs. As shown in Figure 9(a) of the TEM image, different shapes of NPs such as rod and spherical shapes were found. The TEM images have shown the edge of the core and shell NPs. The black area observed in Figure 9(a) is the Co_3_O_4_ core particle surrounded by ZnO shell. In this work, features like core-shell nanostructure arrangement, XRD pattern placement, and morphology were similar to sol-gel synthesized Co_3_O_4_@ZnO core-shell NPs [29].

As depicted in Table 2 (lower panel), the particle size of Co_3_O_4_ core particle was found to be 10.952 ± 0.27 nm. The size of the core material was obtained using the normal (Gaussian) distribution formula mentioned in (5). The value of the size of core particle is less than that of the overall size of the core-shell nanostructure. This indicated that the size of the shell material coating the inner core was larger than the core.

(5) $$\begin{array}{c} Y=y0+\frac{A}{{\left(w\sqrt{\pi /4\;\ln\;\left(2\right)}\right)}^{-4\ln\left(2\right){\left(x-xc\right)}^{2}/w^{2}}}, \end{array}$$

where *y*0 is offset, *xc* is the center of size distribution, *w* is the width of the curve, and *A* is the amplitude of the curve. The normal Gaussian distribution plot in terms of particle size showed that the value of *xc* was 10.68 to 11.22 nm which is the size of the Co_3_O_4_ core nanoparticle.

The average particle size CZCS NPs found from the TEM image was 21.945 nm which is determined under the nonlinear curve Gaussian fit. The particle size obtained from the TEM image was matched with the average crystalline size calculated for X-ray diffraction peaks (22.35 nm).

**Figure 10:**
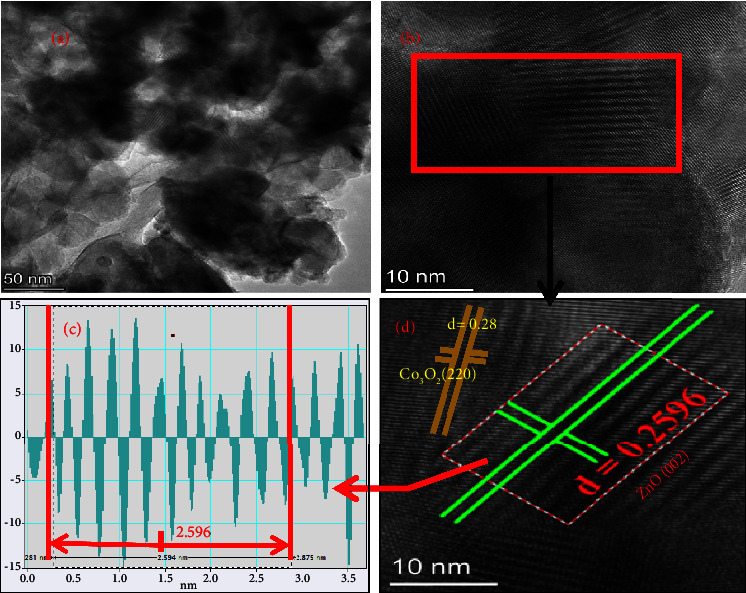
(a) TEM image of biologically synthesized, (b) HR‐TEM, (c) inverted IFFT, and (d) d‐spacing obtained from HR‐TEM Co_3_O_4_@ZnO NPs.

The TEM and HR-TEM analysis explored the grain distribution of synthesized NPs as depicted in Figures 10(a) and 10(b), respectively. The distance between fringes (d-spacing) was determined from HR-TEM using Gatan software. The d-spacing for Co_3_O_4_@ZnO NPs obtained from IFFT of Gatan software and that determined from X-ray diffraction peak using Origin 2023 were almost equal. As indicated in Figures 10(c) and 10(d), the d-spacing for the ZnO (002) and Co_3_O_4_ (220) planes was 0.259 and 0.28 nm, respectively.

**Figure 11:**
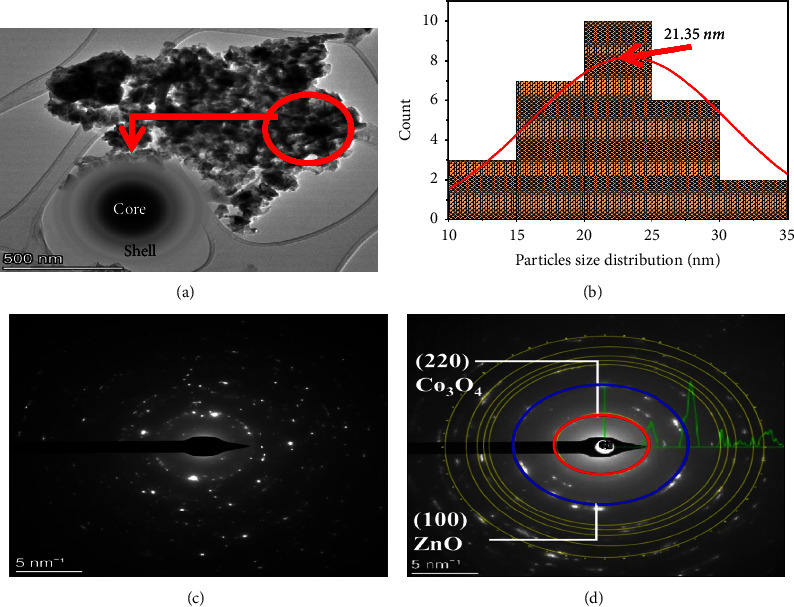
(a) Core-shell image obtained from TEM analysis, (b) histogram graph showing particle size distribution, (c) SAED image, and (d) a number of planes and corresponding Miller index obtained from SAED of Co_3_O_4_@ZnO core-shell NPs.

The crystalline structure of the Co_3_O_4_@ZnO NPs was revealed using selected area electron diffraction (SAED).  Figure 11(c) demonstrates the polycrystalline structure of Co_3_O_4_@ZnO NPs from the SAED image. Unlike that of XRD, electron beams were diffracted from the highly selected area. In SAED analysis, the atoms in the core-shell nanostructure diffracted by the electrons resulted in small bright spots made up of a full ring-like pattern. In XRD analysis, the crystallinity was confirmed by the diffraction peaks of X-ray from the atoms found on the planes, whereas SAED used diffraction of electrons that showed the regularly ordered spots. The bright ring of SAED refers to the fringe alignment which is equivalent to the plane in which its atoms diffract X-ray. The circular electron diffraction depicted in Figure 11(d) has resulted from many crystalline arrangements. In addition to its crystalline nature, SAED is also used for determining the distance between each ring which corresponds to the plane represented by Miller indexes (hkl). The SAED pattern of Co_3_O_4_@ZnO NPs revealed the existence of two rings; the first inner ring with a d-spacing of 0.275 nm and the second with d-spacing value of 0.258 nm. These results are in good agreement with the d-spacing values obtained from XRD analysis attributable to the miller indexes 220 and 002. The plane with miller index 220 belongs to Co_3_O_4_ NPs whereas the 002 plane corresponds to ZnO NPs confirming that Co_3_O_4_ NPs is located at the core and the ZnO NPs exist on the shell of Co_3_O_4_@ZnO core-shell NPs. This investigation indicated good agreement with the core-shell nanostructure formation obtained from TEM image and its particle size distribution shown in Figures 11(a) and 11(b), respectively.

### 3.8. Antibacterial Study

**Figure 12:**
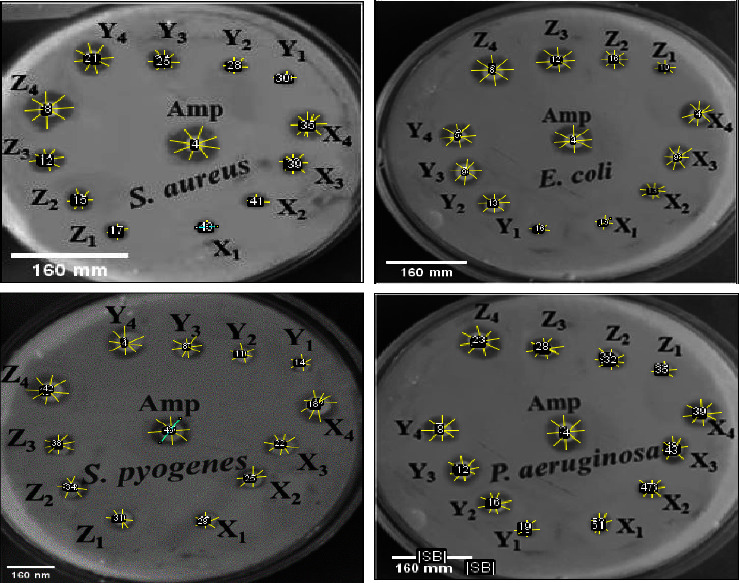
The inhibition zone by biologically synthesized Co_3_O_4_@ZnO core-shell NPs against Gram-negative (*Escherichia coli* and *Pseudomonas aeruginosa*) and Gram-positive (*Staphylococcus aureus* and *Streptococcus pyogenes*) strains.

The inhibition efficiency of Co_3_O_4_@ZnO NPs against Gram-negative (*Escherichia coli* and *Pseudomonas aeruginosa*) and Gram-positive (S*taphylococcus aureus* and S*treptococcus pyogenes*) bacterial strains has been evaluated using the diffusion method.  Table 3 and Figure 12 depict the inhibition zones of the Gram-negative (*S. aureus* and *S. pyogenes*) and Gram-positive (*E. coli* and *P. aeruginosa*) strains by CZCS (1), CZCS (2), and CZCS (3). All three Co_3_O_4_@ZnO core-shell NPs have been applied to the Gram-negative and Gram-positive bacteria within the concentration of 25, 50, 75, and 100 mg/mL. The zone of inhibition was determined by measuring the diameter of the spot four times. The average value of the inhibition zone was taken for each concentration applied to both Gram-positive and Gram-negative bacteria. The antibacterial activities of the Co_3_O_4_@ZnO NPs were found to have increased with increase in the concentration [4].

The highest inhibition zones of CZCS (1) samples against the studied bacterial strains were 17 ± 0.2 (S. aureus), 16 ± 0.32 (S. pyogenes), and 15 ± 0.32 (E. coli), and the CZCS (2) sample exhibited highest inhibition zones of 14 ± 0.32 (P. aeruginosa), 22 ± 0.34 (S. aureus), 13 ± 0.32‐19 ± 0 .32 (S. pyogenes), 10 ± 0.32‐18 ± 0 .45 (E.coli), and 11 ± 0.22-17 ± 0.32 (P. aeruginosa). Similarly, CZCS (3) sample had inhibition zones of 10 ± 0.43‐16 ± 0.38 (S. aureus), 9 ± 0.32‐15 ± 0.43 (S.pyogenes), 8 ± 0.32‐14 ± 0.31 (E. coli), and 9 ± 0.32‐14 ± 0 .43 mm (P. aeruginosa). Among all the samples, CZCS (2) was highly effective even at lower concentration in contrast to the others. The potential activities of CZCS (2) against both Gram-positive and Gram-negative were due to its nanoscale which enables it to penetrate the cell wall of bacteria. The observed differences in the inhibition zones between the Gram-positive and Gram-negative bacteria were believed to be due to the anatomical structural nature of bacterial strains. The small-size core-shell NPs showed high inhibition values even at low concentrations against both Gram-negative and Gram-positive bacteria. As reported in the earlier work [73], the green synthesized Co_3_O_4_@ZnO NPs showed more potent antibacterial activities than individual Co_3_O_4_ and ZnO NPs of the previous study. The red algae extract-mediated Co_3_O_4_ NPs showed good inhibition activity against *S. aureus*, *E. coli*, and *P. aeruginosa* bacterial strains. The report also indicated that the inhibition activity of Co_3_O_4_ NPs increased with the increase in the concentration. Other reports also showed that the antibacterial activities of Co_3_O_4_ and ZnO NPs for *E. coli* which is drug resistant were almost similar with standard drug [73]. However, the novel green synthesized Co_3_O_4_@ZnO NPs in this work inhibit drug-resistant *E. coli* than standard drug. The potent synergistic effect of core and shell was also supported by previous work such that the percentage of dead bacteria at the same concentration by Au@Ag NPs was better than that of their single nanomaterials [6].

In Gram-positive bacteria, the positively charged cobalt and zinc ions from core-shell nanostructures interact with the thick cell wall of bacteria which are negatively charged and cause necrotic damage. In addition to this, the glycerol phosphate and glucosyl phosphate of peptidoglycan which is an anionic polymer trapped by the cobalt and zinc ions of biologically synthesized core-shell nanostructures cleaved the glycoside bond of disaccharide peptide. The main feature that made the Gram-positive bacteria to be susceptible to Co_3_O_4_@ZnO NPs was the absence of an outer membrane [68].

Unlike Gram-positive bacteria, Gram-negative bacteria are highly resistant to antibacterial drugs. However, the small size of Co_3_O_4_@ZnO core-shell NPs showed good inhibitory activities against Gram-negative bacteria than standard drugs (ampicillin). For example, the zone of inhibition measured for CZCS (2) and ampicillin against the drug-resistant *E. coli* was found to be 18 ± 0.45 and 15 ± 0.51 mm, respectively, at 100 mg/mL. However, CZCS (3) and CZCS (1) exhibited inhibition zones of about 14 ± 0.31 and 15 ± 0.32 mm at 100 mg/mL respectively, against *E. coli,* which is less than the standard drug used as control. It has been observed that, at low concentrations, the small-size CZCS (3) showed good performance against Gram-negative bacteria than the large-size CZCS (1). The other reason that Gram-negative bacteria cannot resist the toxicity of Co_3_O_4_@ZnO NPs is the less thickened cell wall, the pore on the outer membrane, and the negatively charged lipopolysaccharide molecules as reported [17, 79].

#### 3.8.1. Mechanistic Interaction of Co_3_O_4_@ZnO NPs

**Figure 13:**
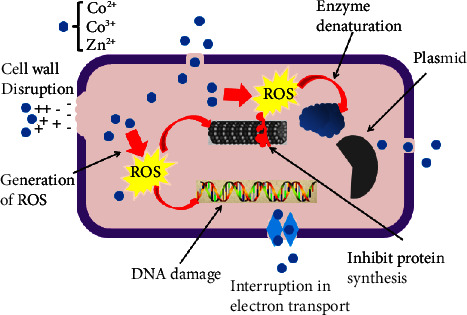
Mechanism of Co_3_O_4_@ZnO NPs action in the bacterial cell.

As reported elsewhere [71], the potential electrical charge of NPs developed the adhesion force which enables it to stick on the surface of the bacterial cell wall. In a similar sense, cobalt and zinc ions from the solution of Co_3_O_4_@ZnO NPs interact with the cell wall of bacteria which causes shrinkage and results in rupturing that leads to cell death. Additionally, the ions of Co_3_O_4_@ZnO NPs bound to the bacterial cell and congested the electron transport chain. On the other hand, as shown in the report of a mechanistic study of Ag NPs [72], the small-size NPs can cross the cell wall of the bacteria. In similar mechanisms, the cobalt and zinc ions of Co_3_O_4_@ZnO NPs can cross the outer membrane through its pore or interact with the thiol groups and deactivated proteins [32]. The Co_3_O_4_@ZnO NPs entered the membrane, which can damage the DNA, deactivate the enzymes, and generate the reactive oxygen species molecules. The free radical oxygen reactive molecules such as hydrogen peroxide (H_2_O_2_), superoxide anion (O_2_^.−^), hydroxyl (HO^.^), peroxyl (RO^.−^), and alkoxy radicals (RO^.^) are generated by ions of Co_3_O_4_@ZnO NPs within the bacterial cell [74, 80]. This reactive oxygen produced by *Datura stramonium* leaf extract-mediated Co_3_O_4_@ZnO NPs caused an oxidative stress which inhibits the growth of both Gram-negative and Gram-positive bacteria as shown in Figure 13.

### 3.9. Antioxidant Study

**Figure 14:**
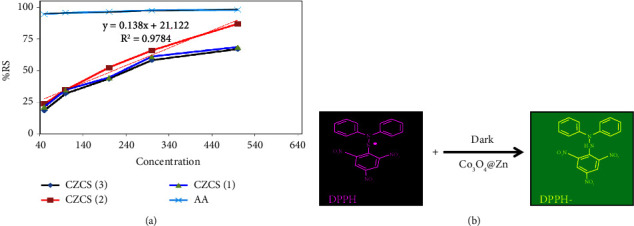
%RSA of (a) CZCS (1), CZCS (2), CZCS (3), and ascorbic acid and (b) reduction mechanism of DPPH.

The antioxidant activity of Co_3_O_4_@ZnO core-shell NPS was measured by utilizing the stable free radical DPPH as shown in Table 4. The addition of methanolic Co_3_O_4_@ZnO NPs to the purple DPPH solution gradually changed to yellow color. In addition to physical visualization, the scavenging capacity of CZCS (1), CZCS (2), and CZCS (3) was determined from the UV-absorbance conducted at 517 nm using (1) mentioned under Section 2.5. As shown in Table 4 and Figure 14(a), the percentage scavenging activities of CZCS (1), CZCS (2), CZCS (3), and ascorbic acid (AA) at 500 *μ*g/mL were found to be 68.63, 86.87, 67.16, and 98.24%, respectively. Among the three ratios, CZCS (2) exhibited a high-efficiency performance in scavenging the DPPH free radical. The half-maximal inhibitory concentration (IC50) of CZCS (2) was also less than that of the other two ratios that assured the good capacity of these NPs at low concentrations. The half-maximal inhibitory concentrations of CZCS (1), CZCS (2), and CZCS (3) were 267.54, 209.26, and 278.2 *μ*g/mL, respectively, which were obtained by using a mathematical linear equation from *Y* = mx ± *c*, where *m* is the slope, *x* is the concentration of the sample, *y* is the percentage scavenging activity, and *c* is constant. The linear regression value also showed a consistent increment of percentage scavenging activity with increasing concentration.

The absorbance of free radicals was decreased at 517 nm as the concentration of Co_3_O_4_@ZnO core-shell NPs increased which increased scavenging capacity. As shown in Figure 14(b), the change in color of DPPH is due to the reduction of the nitrogen atom in the molecule by an electron from the oxygen atom on the Co_3_O_4_@ZnO core-shell NPs. The electron transition is believed to be from n oxygen orbital to antibonding nitrogen orbital (*π*)(*n* ⟶*π*^*∗*^) [19].

## 4. Conclusion

In this work, the Co_3_O_4_@ZnO core-shell NPs were synthesized using three different concentration ratios of core metal to shell precursor salt CZCS (1), CZCS (2), and CZCS (3) with the help of *Datura stramonium* leaf extract as reducing and/or capping agent and calcined at 360°C for 5 h. The formation of all three biologically synthesized core-shell NPs was confirmed using characterization techniques such as FTIR, XRD, UV-DRS, SEM-EDAX, TEM-HRTEM, and SAED. The arrangement of Co_3_O_4_ NPs and ZnO NPs as a core and shell layer, respectively, was confirmed using XRD, TEM, and SAED techniques. The average crystallite sizes of Co_3_O_4_@ZnO core-shell NPs, synthesized within the concentration ratios CZCS (1), CZCS (2), and CZCS (3), were found to be 25 ± 1.4, 22 ± 1.5, and 25 ± 1.5 nm, respectively. Among the three Co_3_O_4_@ZnO core-shell NPs, CZCS (2) possess a small size (22.35 nm) and large band gap energy (2.76 eV). The particle size of core particle is 10.952 ± 0.27 nm which is less than half of the average crystallite size of core-shell NPs, and this was matched with data from a previous study in which the size of the core must be less than the size of shell NPs. The rod-like shape and spherical shape of Co_3_O_4_@ZnO NPs were revealed from SEM and TEM characterization techniques. In this study, the high inhibition performance of Co_3_O_4_@ZnO NPs was evaluated against Gram-positive and Gram-negative bacteria. The average inhibition zones of nanosize CZCS (2) at 100 mg/mL against *S. aureus*, *S. pyogenes*, *E. coli*, and *P. aeruginosa* were found to be 22 ± 0.34, 19 ± 0.32, 18 ± 0.45, and 17 ± 0.32 mm, respectively, which are greater than those of standard drug ampicillin. The high-performance antibacterial activities of core-shell NPs against Gram-positive and Gram-negative pathogens are due to the synergistic effect developed from the Co_3_O_4_ core and ZnO shell structures. Similarly, the small-size CZCS (2) showed 86.87% scavenging capacity and IC50 of 209.26 *μ*g/mL. The synthesized Co_3_O_4_@ZnO NPs have the potentiality to be better antibacterial and antioxidant material. Finally, we conclude that the Co_3_O_4_@ZnO NPs were biologically synthesized and showed good antibacterial activities against drug-resistant human pathogens which are a public health challenge worldwide.

## Figures and Tables

**Table 1:** Some selected phytochemicals screened from *Datura stramonium* leaf extract.

S.No.	Phytochemicals	Reagents	Result
1	Alkaloids	Wagner's	+
2	Flavonoids	Alkaline	+
3	Tannins	Alkaline	+
4	Saponins	Frothing	+
5	Phenol	FeCl_3_	+
6	Phytosterols	Salkowski	+
7	Glycosides	Salkowski	+
8	Steroids	Burchard	−
9	Terpenoids	Salkowski	+
10	Anthraquinones	Borntrager	+

**Table 2:** The particle size distribution of Co_3_O_4_ core particle (lower panel) and the d-spacing of CZCS obtained from XRD patterns and HR-TEM images (upper panel).

Nanoparticles	2*θ*	d-spacing standard	d-spacing (XRD)	d-spacing (HR-TEM)	(hkl)
Co_3_O_4_	31.85	2.8114	2.8075	2.802	220
ZnO	34.54	2.5958	2.59503	2.594	002
ZnO	36.34	2.4738	2.4697	2.5	101
ZnO	47.65	1.9064	1.9073	1.87	012
ZnO	56.68	1.6248	1.623	1.632	110
ZnO	62.98	1.4755	1.475	1.466	122
Co_3_O_4_	68.06	1.3772	1.376	1.3677	440

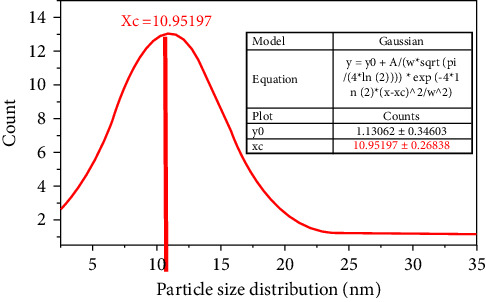					

**Table 3:** The inhibition zone of Co_3_O_4_@ZnO core-shell NPs against bacterial strain.

NPs	Conc. (mg/mL)	Gram-positive				Gram-negative			
		*S. aureus* ATCC25923		*S. pyogenes* ATCC19615		*E .coli* ATCC25922		*P. aeruginosa* ATCC27853	
		Avg	StDv	Avg	StDv	Avg	StDv	Avg	StDv
CZCS (1)	*X*_4_ = 100	17 ± 0.2	0.408	16 ± 0.32	0.645	15 ± 0.32	0.645	14 ± 0.32	0.645
	*X*_3_ = 75	15 ± 0.32	0.645	15 ± 0.32	0.645	14 ± 0.43	0.854	12 ± 0.31	0.866
	*X*_2_ = 50	13 ± 0.24	0.479	12 ± 0.32	0.645	12 ± 0.2	0.408	11 ± 0.3	0.624
	*X*_1_ = 25	10 ± 0.41	0.826	10 ± 0.46	0.913	9 ± 0.27	0.538	9 ± 0.27	0.645

CZCS (2)	*Z*_4_ = 100	22 ± 0.34	0.685	19 ± 0.32	0.645	18 ± 0.45	0.909	17 ± 0.32	0.645
	*Z*_3_ = 75	18 ± 0.43	0.854	17 ± 0.27	0.538	15 ± 0.32	0.645	14 ± 0.43	0.866
	*Z*_2_ = 50	15 ± 0.32	0.645	14 ± 0.46	0.913	12 ± 0.24	0.473	12 ± 0.24	0.479
	*Z*_1_ = 25	12 ± 0.24	0.479	13 ± 0.32	0.645	10 ± 0.32	0.645	11 ± 0.22	0.435

CZCS (3)	*Y*_4_ = 100	16 ± 0.38	0.75	15 ± 0.43	0.854	14 ± 0.31	0.629	14 ± 0.43	0.854
	*Y*_3_ = 75	15 ± 0.43	0.854	14 ± 0.24	0.479	13 ± 0.47	0.946	13 ± 0.34	0.678
	*Y*_2_ = 50	14 ± 0.32	0.645	13 ± 0.35	0.707	10 ± 0.46	0.913	11 ± 0.43	0.854
	*Y*_1_ = 25	10 ± 0.43	0.854	9 ± 0.32	0.645	8 ± 0.32	0.645	9 ± 0.32	0.645

Amp	100	17 ± 0.8	0.798	16 ± 0.47	0.949	15 ± 0.51	0.898	15 ± 0.43	0.86

Amp is ampicillin, Avg is average, and StDv is standard deviation.

**Table 4:** Percentage radical scavenging and IC50 of Co_3_O_4_@ZnO core-shell NPs.

Conc. (*μ*g/mL)	Co_3_O_4_@ZnO core-shell NPs						Ascorbic acid	
	CZCS (1)		CZCS (2)		CZCS (3)			
	Abs	%RSA	Abs	%RSA	Abs	%RSA	Abs	%RSA
50	0.533	21.57	0.5167	24.02	0.5533	18.63	0.036	94.71
100	0.4433	34.80	0.4433	34.80	0.4633	31.86	0.028	95.88
200	0.3767	44.60	0.3233	52.45	0.3833	43.63	0.024	96.47
300	0.2633	61.27	0.230	66.18	0.2833	58.33	0.016	97.65
500	0.2133	68.63	0.089	86.87	0.2233	67.16	0.012	98.24
IC50	267.54		209.26		278.2			

## Data Availability

The data used to support the findings of this study are available from the corresponding authors upon request.
